# Supplementary figures and images for: The Known and Unknown of Global Tick-Borne Viruses
Source: Viruses. 2024 Nov 21;16(12):1807. doi: 10.3390/v16121807 (PMC11680321; doi:10.3390/v16121807)

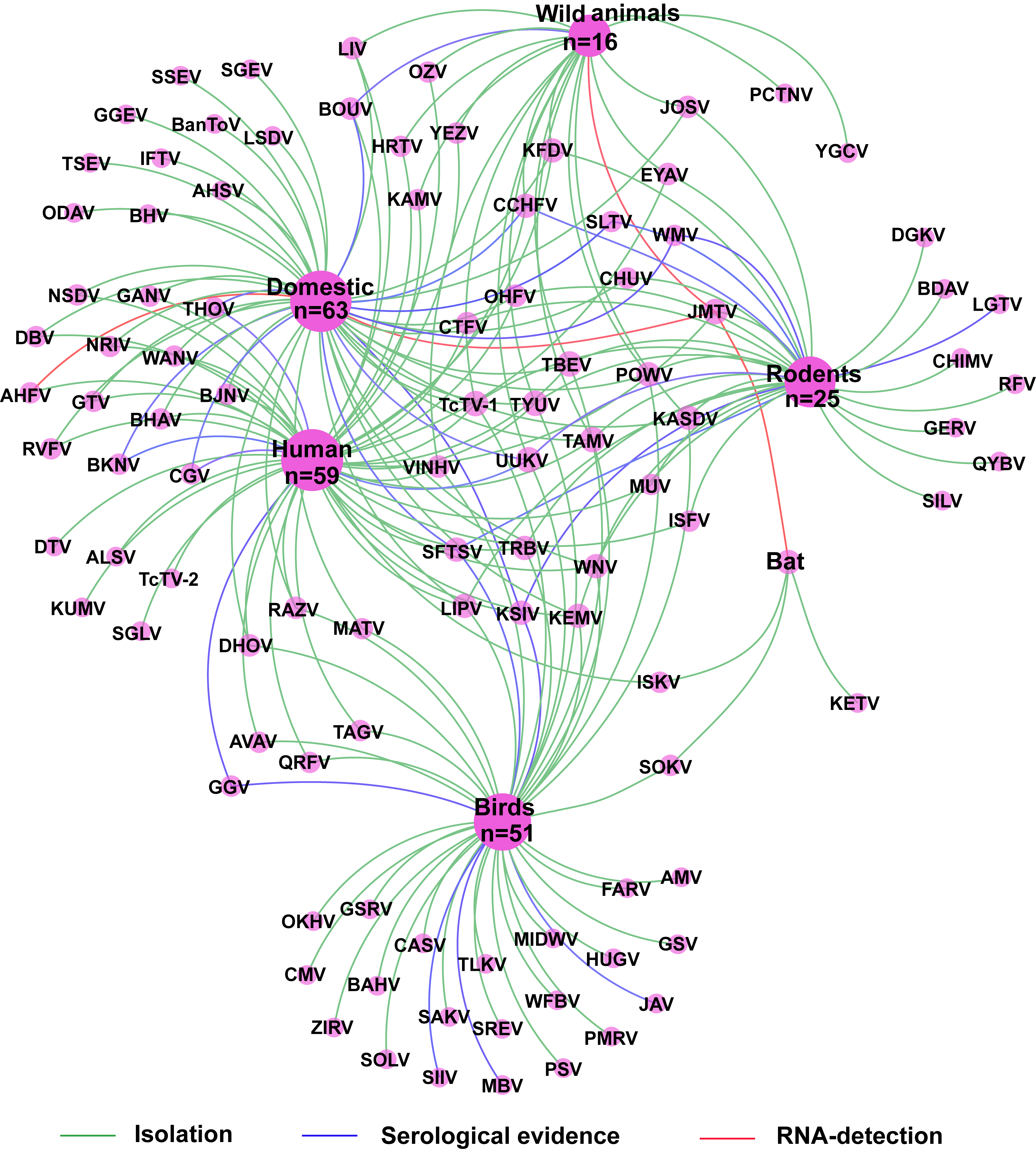

Supplement: Supplementary file 1 [file viruses-16-01807-s001.zip › Supplementary Figure S1.tif]
